# Menstruation among In-School Adolescent Girls and Its Literacy and Practices in Nigeria: A Systematic Review

**DOI:** 10.3390/medicina59122073

**Published:** 2023-11-24

**Authors:** Chinomso Adanma Uzoechi, Ali Davod Parsa, Ilias Mahmud, Ibrahim Alasqah, Russell Kabir

**Affiliations:** 1 School of Allied Health, Faculty of Health, Medicine and Social Care, Anglia Ruskin University, Chelmsford CM1 1SQ, UK; cuzoechi1290@gmail.com (C.A.U.); ali.parsa@aru.ac.uk (A.D.P.); russell.kabir@aru.ac.uk (R.K.); 2 School of Health, University of New England, Armidale, NSW 2351, Australia; imahmud@une.edu.au; 3 BRAC James P Grant School of Public Health, BRAC University, Dhaka 1212, Bangladesh; 4 Department of Public Health, College of Public Health and Health Informatics, Qassim University, Al Bukairiyah 52571, Saudi Arabia

**Keywords:** menstruation, adolescent, menarche, menstrual literacy, menstrual cycle, school, systematic review

## Abstract

*Background and Objectives:* Menstruation is a natural occurrence marked by the periodic release of endometrial cells within the uterine lining from the female genital area. Menstruation knowledge remains highly essential for young adolescents. Inadequate awareness and understanding of menstruation have far-reaching consequences on the overall wellbeing and health outcomes of young adults worldwide. Adolescent girls make up a large percentage of high school students in Nigeria. Girls in countries with low to middle incomes are frequently misled or uneducated regarding menstruation. Menstrual health literacy (MHL) is the level of knowledge concerning matters related to menstrual health. It is observed that a lack of menstrual health literacy is seen among young adults. This systematic review aimed to examine menstruation literacy, attitudes, and adolescent girls’ practices in Nigeria. *Materials and Methods:* This systematic review included quantitative, cross-sectional, quasi-experimental, and qualitative primary research studies relating to menstruation literacy, attitudes, and practices of adolescents in Nigeria. Articles for this study were searched for on databases such as PubMed and BioMed Central using keywords. These studies were subjected to stringent inclusion and exclusion criteria where the Preferred Reporting Items for Systematic Reviews and Meta-Analyses (PRISMA) guidelines were used, and 13 articles were included after critical appraisal. Data extracted were analysed using narrative synthesis. *Results:* Findings indicated that knowledge regarding menstruation among adolescents (82.6%) was poor. Menstruation information was obtained from mothers, which was positive as some adolescents reported their closeness to their mothers. Regarding attitudes towards menstruation among adolescents, it was reported that more respondents (70.3%) had negative attitudes towards menstruation. *Conclusions:* Most of the respondents in Nigeria were not adequately prepared for the onset of their first menstrual period. Knowledge and attitude levels were low regarding periods for adolescents. The only exception was their positive attitude towards using water and soap to wash their hands during menstruation. The review shows a significant gap between adolescents’ menstruation knowledge and actual hygienic methods during menstruation. It is therefore required for educational awareness programmes and campaigns to be put in place to educate adolescents about menstruation.

## 1. Introduction

Menstruation is a natural occurrence characterised by the periodic release of blood cells and mucous cells from the uterine lining through the vagina. Blood cells such as red blood cells or erythrocytes transport oxygen from the lungs to tissues and organs throughout the body and transport carbon dioxide back to the lungs for exhalation. Other cells include white blood cells and platelets. Mucous cells are specialised cells that produce mucus, a thick and slippery fluid that serves as protection in different parts of the body [1]. This is the first sign that a woman has started her reproductive years. It usually lasts between two and seven days. Menarche first appears between the ages of puberty and adolescence and is the defining event of the teenage female experience [2].

Adolescence is defined as the years of 10–19, when a person goes through a time of fast development from childhood to maturity [3]. About 20% of the population globally are adolescents, the vast majority of whom are in less-developed nations. In Nigeria, adolescent girls make up a large percentage of high school students. They make up a substantial percentage of the nation’s total population (44.8%) and experience several issues that could be harmful to their health [4,5].

Globally, there are around 1.2 billion teenagers in the world. About 25% of Nigerian children do not have access to private spaces for excrement and handling their menstrual cycles [6]. This has resulted in almost 10 million children, predominantly females, dropping out of school.

Menstrual health knowledge or literacy is especially essential for school-aged individuals, as the teenage period begins throughout this period. Girls in countries with low to middle incomes (LMICs) are frequently misled or uneducated regarding sexual and reproductive health knowledge, leaving them unprepared for menstruation [7]. Through periods, ignorance can contribute to humiliation, loneliness, and unclean practices. A large proportion of young women do not have a comprehension of menstruating and maturation [8]. For menstrual hygiene knowledge, the emphasis is on sharing facts about how to educate adolescent females on the proper and clean management of menstruation [9]. Menstrual health literacy (MHL) is the level of knowledge concerning matters related to menstrual health. Within this context, it is observed that there is a deficiency in menstrual health literacy among in-school adolescents [10].

Menstrual health literacy also refers to an individual’s knowledge and understanding of various aspects related to menstruation. Improving menstrual health literacy is important for promoting overall wellbeing, reducing stigma, and ensuring that individuals can manage their menstrual health effectively and safely [11]. It is a critical component of sexual and reproductive health education, as it empowers individuals, especially young girls, to make decisions regarding their menstrual health. It helps individuals recognise normal menstrual patterns, understand the importance of menstrual hygiene, and address any menstrual-related challenges or issues they may encounter [12,13].

Knowledge can be understood as the amalgamation of our conceptualisations, perspectives, and propositions. In the present framework, knowledge refers to the comprehension derived from subjective experiences, pertaining specifically to the practical aspects of menstruation as observed in females. In the context of developing nations, there exists a prevalent cultural phenomenon characterised by a reluctance to openly discuss menstruation and its associated matters. Consequently, a significant number of adolescent females are deficient in adequate knowledge regarding menstrual hygiene. The available literature suggests that a significant proportion of adolescent females possess inadequate and wrong knowledge regarding the physiological aspects and hygiene practices associated with menstruation [14,15,16]. Research conducted in Africa has indicated a notable deficiency in overall awareness and understanding of menstruation, as evidenced by the findings reported [17]. Research conducted on Nigerian schoolgirls has revealed that a significant proportion, ranging from 31% to 56%, employ sanitary materials as a means to collect blood from their periods, rather than utilising sanitary pads [18]. The absence of adequate knowledge regarding secure and clean menstrual practices among adolescent girls represents a significant gap in knowledge and an unfulfilled requirement for sexuality education [19].

The term “attitude” refers to the evident ethical behaviour of adolescent females concerning menstruation in the context of this research study [20]. The attitudes about hygiene during menstruation (MH) are referred to as menstrual sanitation behaviours. Attitudes can exhibit either positive or negative characteristics, thereby exerting an influence on an individual’s overall wellbeing or potentially contributing to the occurrence of reproductive tract infections. It was determined that adolescents exhibit unfavourable attitudes regarding hygiene during menstrual periods [21].

Fostering a positive attitude to menstruation and promoting good menstrual hygiene practices can effectively protect the health of adolescents by mitigating their susceptibility to reproductive infections [22]. In the Nigerian context, it is commonly observed that adolescents frequently react to menstruation as an indication of illness. Adolescent girls tend to view menstruation as a source of embarrassment that should be concealed [23].

Inadequate adherence to menstrual hygiene practices has the potential to give rise to significant health hazards, such as sexual and urinary tract illnesses, which may subsequently lead to birth and infertility issues during childbirth [24]. The issue of menstrual hygiene among adolescents in Nigeria has not been adequately addressed, leading to increased susceptibility to sexual diseases, abdominal inflammatory disorders, and related difficulties due to unsafe practices during menstruation [25,26]. In Southeast and Northwest Nigeria, a study was conducted revealing that a significant proportion of adolescent school girls, specifically 64% and 77% in the respective regions, lacked education before menstruation. This absence of menstruation knowledge led to unfavourable menstrual experiences and inadequate menstrual hygiene practices among these individuals [15].

The research question for this review is “What is the menstruation literacy and practices among adolescent girls in Nigeria?” This systematic review aimed to explore associated factors with menstruation among adolescents in Nigeria. The objective of this systematic review was to investigate menstruation literacy, attitudes, and practices of adolescent girls and identify consequences associated with poor menstrual literacy in Nigeria. This review provides a comprehensive analysis of the existing body of literature on the topic of adolescent menstrual health literacy, specifically focusing on the knowledge, attitude, and practices related to menstruation hygiene, within the context of Nigeria.

## 2. Methods

### 2.1. Study Design

This systematic review included quantitative, cross-sectional, quasi-experimental, and qualitative primary research studies relating to menstruation literacy, attitudes, and practices of adolescents in Nigeria.

### 2.2. Search Strategy

The review was conducted using a comprehensive search for articles related to this topic on various academic databases, including EBSCO, CINAHL Plus, PubMed, BioMed Central, and EMBASE. The search was performed using relevant keywords. Subsequently, a search was conducted within the Cochrane Database of Systematic Reviews (CDSR) to identify any preexisting or continuing systematic reviews. Several systematic reviews were identified on the topic of menstruation literacy; however, no systematic review specifically addressing the research title was located. The systematic reviews employed the Preferred Reporting Items for Systematic Reviews and Meta-Analyses (PRISMA) standards to conduct a thorough search of the published literature to find various articles. This study is in accordance with the most recent Preferred Reporting Items for Systematic Reviews and Meta-Analyses Statement (PRISMA 2020) guidelines for reporting systematic reviews of healthcare interventions.

The scope of the literature search was restricted to the geographical region of Nigeria, while the temporal scope was not confined to a specific year. Multiple databases were utilised to mitigate the risk of overlooking significant research and to reduce potential bias. The review utilised the SPIDER search strategy tools, which consist of Sample, Phenomenon of Interest, Design, Evaluation, and Research type, to locate appropriate keywords. These tools were chosen for their appropriateness in determining inclusion and exclusion criteria (see Table 1).

The utilisation of Boolean operators, namely conjunctions, was employed to attain outcomes that were more targeted. Additionally, the indexing of publications was facilitated through the implementation of Medical Subject Headings (MeSH) keywords. They include “menstrual health literacy in adolescents”, “menstrual health literacy”, “menstrual health education”, “menstrual hygiene management in schools”, “menstrual health in schools”, “menstruation in schools and menstrual education in schools”, “intervention”, “school-based”, “adolescents”, “education”, “programs”, “menstruation health”, and “knowledge, attitudinal disposition and practices regarding menstruation and Nigeria”. Search term 1 “adolescent” OR “girl” OR “teenage” AND “youth” OR “young” OR “pre-adolescent” OR “school-girls” OR “in-school adolescents” AND “female” OR “woman” AND Search term 2 “Menstruation” AND “menarche” OR “menses” OR “menarche” OR “menstrual health” OR “menstrual hygiene” OR “menstrual management” OR “dysmenorrhea” OR “sanitation” OR “menstrual etiquette” AND Search term 3 “Menstrual health literacy” OR “information” OR “knowledge” OR “menstruation health education” OR “literacy” AND Search term 4 “attitude” AND “practice” AND Search term 5, “West Africa” OR “Nigeria”.

**Figure 1:**
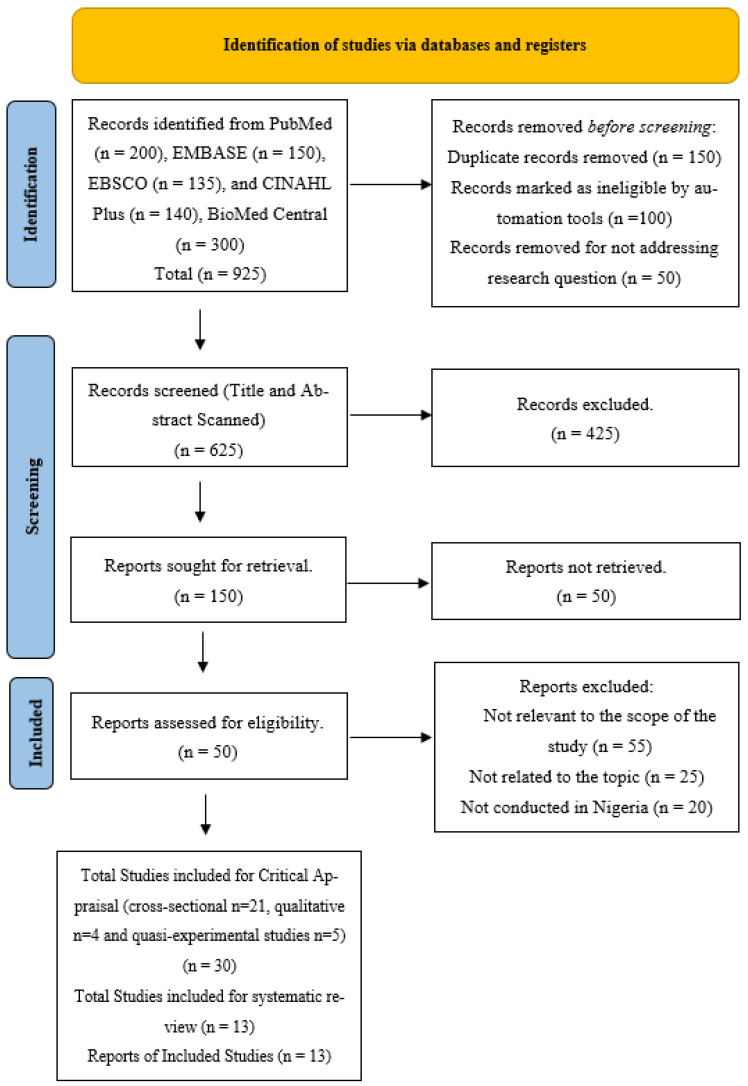
Preferred Reporting Items for Systematic Reviews and Meta-Analyses (PRISMA) 2020 flow diagram.

The review applied search limitations to narrow down the focus of the search to the main peer-reviewed papers that were accessible in English and available in full-text format (see Figure 1). Our literature search records are presented in Table 2.

### 2.3. Study Selection

The inclusion and exclusion criteria are listed in Table 3.

The publications included in the analysis are indicative of the comprehensive body of research that has been undertaken and subsequently published. The researchers utilised Google Scholar to conduct a comprehensive search for scholarly publications and conference abstracts that were not yet incorporated in their study. This search exclusively incorporated peer-reviewed publications due to their provision of research of superior quality, hence reducing the potential for bias.

### 2.4. Inclusion Criteria and Exclusion Criteria

In the initial phase, the papers obtained through the search were evaluated based on their study design after applying the specified limitations. During the second phase, the search results were examined for titles and abstracts that specifically mentioned sample and study designs focused on data collection through questionnaires and interviews encompassing qualitative, cross-sectional, and intervention research methodologies. During the third stage, the implementation of articles focused on examining the phenomenon of interest and its corresponding effect. During the fourth stage of the study, a comprehensive search was conducted to identify full-text articles that were pertinent to the topic of menstruation. These articles were then carefully examined to extract important data related to the phenomenon of interest and the desired outcome. Excluded from consideration were those that presented inadequate or lacking information about menstruation, or those that lacked a clear focus on the topic. Following the application of the predetermined inclusion and exclusion criteria, a total of 30 scholarly publications were selected for the subsequent critical evaluation phase (see Figure 1 and Table 3).

### 2.5. Critical Appraisal

The process of critical evaluation was conducted on a total of 30 papers, wherein an examination was made of the methodological strengths and weaknesses of each study, the validity of the research conducted, the trustworthiness of the obtained results, and the potential presence of biases. Additionally, this was conducted to assess the adequacy of the study design, implementation, and reporting, as well as to determine if the findings contribute substantively to addressing the research issue posed in this systematic review. Various appraisal tools were utilised to evaluate the studies. The Critical Appraisal Skills Programme (CASP) tool was utilised to evaluate qualitative studies (see Table 4). The Appraisal tool for Cross-Sectional Studies (AXIS), specifically designed for assessing this study design, was employed to appraise cross-sectional studies (see Table 5). The Joanna Briggs Institute (JBI) checklist for quasi-experimental studies was utilised to evaluate quasi-experimental studies (see Table 6). The ethical assessment conducted in this review aimed to enhance the ethical and methodological rigour by excluding studies that had ethical deficiencies.

#### 2.5.1. Critical Appraisal and Ethical Appraisal Outcome

After conducting a critical and ethical evaluation, a total of 13 papers were deemed suitable for inclusion in the review. A total of 17 studies were excluded from the systematic review due to various reasons. These reasons include the studies’ low internal validity, which compromises the reliability of their findings, as well as their low validity and reliability overall. Additionally, exclusion was also based on the absence of ethical considerations, such as insufficient information regarding permission from participants, confidentiality, and the acquisition of ethical clearance.

#### 2.5.2. Data Abstraction

The data were collected through the utilisation of a table generated in Microsoft Word. The retrieved data encompassed several elements, such as the textual reference of the article, the study location, the methodology of the study, the number of samples, the intent of the investigation, the results of the study, and the constraints of the study.

### 2.6. Data Analysis

This systematic review incorporated data from both quantitative research and quasi-experimental investigations, precluding the possibility of carrying out a meta-analysis. The data taken from the included papers were organised and analysed using Microsoft Excel. The research methodology employed for this study was a meta-synthesis, specifically a textual narrative synthesis. This is because of the nature and heterogeneity of the studies, which include different study designs, sample sizes and methodology, different types of studies, and selection bias. Narrative synthesis denotes a method for systematically reviewing and amalgamating results from multiple studies, primarily utilising words and text to encapsulate and elucidate the synthesis’s findings. Text-based narrative synthesis enhances the understanding of each study’s context and distinctive attributes.

Meta-analysis was not conducted because the included studies varied significantly in terms of methodology, study designs, and outcome measures, which summed up heterogeneity, and this can limit the validity and reliability of conducting a meta-analysis. There was also a limited number of studies available on the topic in regard to the country of study where conducting a meta-analysis may not be feasible or may not provide sufficient statistical power. A narrative synthesis was then used to summarise existing evidence.

Textual narrative synthesis is a method that categorises studies into the same groups. This approach has been effective in integrating evidence from diverse sources such as qualitative and quantitative studies. Textual narrative synthesis is particularly adept at revealing variations between studies, and it addresses issues related to quality assessment. This is achievable because textual narrative synthesis enhances understanding of each study’s context and unique attributes [27].

**Table 4:** Critical appraisal for qualitative studies using the Critical Appraisal Skills Programme (CASP) tool.

Qualitative Studies: CASP Tool				Section A: Are the Results Valid?				Section B: What Are the Results?		
Reference	Was There a Clear Statement of the Aims of the Research?	Is a Qualitative Methodology Appropriate?	Was the Research Design Appropriate to Address the Aims of the Research?	Was the Recruitment Strategy Appropriate to the Aims of the Research?	Were the Data Collected in a Way that Addressed the Research Issue?	Has the Relationship between the Researcher and Participants Been Adequately Considered?	Have Ethical Issues Been Taken into Consideration?	Was the Data Analysis Sufficiently Rigorous?	Is There a Clear Statement of Findings?	How Valuable is the Research?
Tomlinson 2022 [28]	+	+	+	+	+	+	+	+	+	+
Obioma et al., 2015 [29]	+	+	+	+	+	+	+	+	+	+
Salau and Ogunfowokan 2017 [30]	+	+	+	+	+	+	+	+	+	+
Adegbayi 2017 [31]	+	+	+	+	+	+	+	+	+	+

(+) = Item adequately addressed.

**Table 5:** Critical appraisal for cross-sectional studies using the Appraisal tool for Cross-Sectional Studies (AXIS).

		Introduction					Methods			
Reference	Were the Aims/Objectives of the Study Clear?	Was the Study Design Appropriate for the Stated Aim(s)?	Was the Sample Size Justified?	Was the Target/Reference Population Clearly Defined? (Is It Clear Who the Research Was About?)	Was the Sample Frame Taken from an Appropriate Population Base So That It Closely Represented the Target/Reference Population under Investigation?	Was the Selection Process Likely to Select Subjects/Participants That Were Representative of the Target/Reference Population under Investigation?	Were Measures Undertaken to Address and Categorise Non-Responders?	Were the Risk Factor and Outcome Variables Measured Appropriate to the Aims of the Study?	Were the Risk Factor and Outcome Variables Measured Correctly Using Instruments/Measurements That Had Been Trialled, Piloted, or Published Previously?	Is It Clear What was Used to Determine Statistical Significance and/or Precision Estimates? (e.g., p-Values, Confidence Intervals)
Rasheed and Afolabi 2021 [26]	+	+	+	+	+	+	+	+	+	+
Gorah, Haruna and Ufwil 2020 [20]	+	+	+	+	+	+	+	+	+	+
Jimin et al., 2023 [5]	+	+	+	+	+	+	+	+	+	+
Fehintola et al., 2017 [18]	+	+	+	+	+/-	+/-	+	+	+	+
Edet et al., 2020 [19]	+	+	+/-	+	+	+	-	+	+	+
Umahi et al., 2021 [15]	+	+	+	+	+	+	+	+	+/-	+
Okafor-Terver & Chuemchit, 2017 [32]	+	+	+	+	+	+	+	+/-	+	+
Nkemdilim, Nwosu and Chris 2023 [33]	+	+	+	+	+	+	+/-	+	+/-	+
Nwimo et al., 2022 [34]	+	+	+	+	+	+	+	+	+	+/-
Emmanuel and Amadaowei 2020 [35]	+	+	+	+	+	+	+	+/-	+	+
Isah, Ibrahim and Aminu 2022 [36]	+	+	+	+	+	+	+	+	-	+
Ilo, Nwimo and Chinagorom 2016 [4]	+	+	+	+	+	+	+	-	+	+
Bolanle, Ayoade and Sola 2021 [37]	+	+	+	+	+	+	+	+	+	+
Ekoko and Ikolo 2021 [9]	+	+	+	+	+	+	+/-	+	-	+
Buradum, Etor and Edison 2020 [38]	+	+	+	+	+	+	+	+	+	+
Popoola et al., 2021 [39]	+	+	+	+	+	+	+	+	+	+
Obande-Ogbuinya et al., 2022 [23]	+	+	+	+	+	+	+	+/-	+	+
Idoko et al., 2022 [40]	+	+	+	+	+	+	+	+	+	+
Okeke et al., 2021 [41]	+	+	+	+	+	+	+/-	+	+	+
Garba, Rabiu and Abubakar 2018 [22]	+	+	+	+	+	+	+	+	+	+
Ibeagha 2022 [21]	+	+	+	+	+	+	+	+	+	+
				**Results**			**Discussion**		**Others**	
**Reference**	**Were the Methods (Including Statistical Methods) Sufficiently Described to Enable Them to Be Repeated?**	**Were the Basic Data Adequately Described?**	**Does the Response Rate Raise Concerns about Non-Response Bias?**	**If Appropriate, Was Information about Non-Responders Described?**	**Were the Results Internally Consistent?**	**Were the Results Presented for All the Analyses Described in the Methods?**	**Were the Authors’ Discussions and Conclusions Justified by the Results?**	**Were the Limitations of the Study Discussed?**	**Were There Any Funding Sources or Conflicts of Interest that May Affect the Authors’ Interpretation of the Results?**	**Was Ethical Approval or Consent of Participants Obtained?**
Rasheed and Afolabi 2021 [26]	+/-	+/-	+/-	+/-	+/-	+/-	+/-	+/-	+	+
Gorah, Haruna and Ufwil 2020 [20]	+	+	+/-	+	+	+	+	+	+	+
Jimin et al., 2023 [5]	+	+	-	+	+	+	+	+/-	+	+
Fehintola et al., 2017 [18]	+	+	+/-	+	+	+	+	+	+	+
Edet et al., 2020 [19]	+	+	+/-	-	+	+	+	+	-	+
Umahi et al., 2021 [15]	+	+	-	+	+	+	+/-	-	+	+
Okafor-Terver & Chuemchit, 2017 [32]	+	+	+	+	+	+	+	+	+	+
Nkemdilim, Nwosu and Chris 2023 [33]	+	+	+	+	+	+	+	+/-	+	+
Nwimo et al., 2022 [34]	+	+	+	+	+	+	+	+	+	+
Emmanuel and Amadaowei 2020 [35]	+	+	+	+	+	+	-	+	+	+
Isah, Ibrahim and Aminu 2022 [36]	+	+	+	+	+/-	+	+	+/-	+	+
Ilo, Nwimo and Chinagorom 2016 [4]	+	+	+	+	+	+	+	+	+	+
Bolanle, Ayoade and Sola 2021 [37]	+	+	+	+	+	+	+	+/-	+	+
Ekoko and Ikolo 2021 [9]	+	+	+	+	+	+/-	+	+	+	+
Buradum, Etor and Edison 2020 [38]	+	+	+	+	+	+	+	+	+/-	+
Popoola et al., 2021 [39]	+	+	+	+	+	+	+	+/-	+	+
Obande-Ogbuinya et al., 2022 [23]	+	+	+	+	+	+	-	+	+	+
Idoko et al., 2022 [40]	+	+	+	+	+	+	+	+	+	+
Okeke et al., 2021 [41]	+	+	+	+	+	+	+	+	+	+/-
Garba, Rabiu and Abubakar 2018 [22]	+	+	+	+	+	+	+	+/-	+	+
Ibeagha 2022 [21]	+	+	+	+	+	+	+	+	+	+

(+) = Item adequately addressed; (-) = item not adequately addressed; (+/-) = item partially addressed.

**Table 6:** Results of critical appraisal of quasi-experimental studies.

JBI Checklist Criteria (Potential Bias and Threat)	Studies				
	Ogunleye and Kio 2020 [2]	Agbede and Ekeanyanwu 2021 [6]	Chinasa and Catherine 2021 [42]	Adegoke and Janet 2022 [43]	Omovie, Agbapuonwu and Makata 2021 [44]
1. Is it clear in the study what is the ‘cause’ and what is the ‘effect’ (i.e., there is no confusion about which variable comes first)?	No (-)	Yes (+)	Yes (+)	Yes (+)	Yes (+)
2. Were the participants included in any comparisons similar?	Yes (+)	Yes (+)	Yes (+)	Yes (+)	Yes (+)
3. Were the participants included in any comparisons receiving similar treatment/care, other than the exposure or intervention of interest?	Yes (+)	Yes (+)	Yes (+)	Yes (+)	Yes (+)
4. Was there a control group?	Yes (+)	Yes (+)	Yes (+)	Yes (+)	Yes (+)
5. Were there multiple measurements of the outcome both pre- and post-intervention/exposure?	Yes (+)	Yes (+)	Yes (+)	Yes (+)	Yes (+)
6. Was follow-up complete and, if not, were differences between groups in terms of their follow-up adequately described and analysed?	No (-)	Yes (+)	Yes (+)	Yes (+)	No (-)
7. Were the outcomes of participants included in any comparisons measured in the same way?	No (-)	Yes (+)	No (-)	Yes (+)	No (-)
8. Were outcomes measured reliably?	Yes (+)	Yes (+)	Yes (+)	Yes (+)	Yes (+)
9. Was appropriate statistical analysis used?	Yes (+)	Yes (+)	Yes (+)	Yes (+)	Yes (+)
Total (%) and quality rating	8/9 (88%) Good	8/9 (88%) Good	8/9 (88%) Good	8/9 (88%) Good	8/9 (88%) Good

Good: at least 80%; moderate: 50–80%; poor: less than 50%.

## 3. Results

### 3.1. Included Study Characteristics

The review encompassed a total of thirteen pieces of research conducted in various regions of Nigeria. The mixed-method investigations were undertaken in the southern and western regions of Nigeria. The cross-sectional study was carried out in the western area of Nigeria. The present study utilised cross-sectional research conducted in several regions of Nigeria, including the western, southern, eastern, and northern areas. All the research investigations were conducted only among adolescents attending schools in Nigeria. The primary objective of eleven studies was to provide a comprehensive depiction of the knowledge, attitudes, and practices about menstruation among adolescent females. Some articles investigated one or two of the variables such as knowledge only, attitude regarding menstruation only, and both knowledge and attitude respectively. One study examined sources of premenstrual information and practices during the menstrual period. The primary objective of one study was to assess the impact of an educational programme on menstrual hygiene practices.

### 3.2. Included Study Designs

There were two mixed-method studies which included qualitative and quantitative methods in which data were collected using in-depth interviews and questionnaires. There was one quasi-experimental study where there was an educational intervention for adolescents and data were collected using a structured questionnaire. Lastly, the review had ten cross-sectional studies where information was gathered utilising validated questionnaires.

### 3.3. Information Regarding Knowledge, Attitude, and Practices Regarding Menstruation

The source of data obtained from all studies included in this review was the respondents who participated in the research. The studies also had samples from age groups between 10 and 19 years of age who were female adolescents and attending school. Table 7 presents comprehensive data encompassing the study setting, sample size, study findings, and study limitations.

### 3.4. Summary of Findings

The results of the studies included in the review showed multiple related patterns and similar findings. Additionally, most of the studies featured a cross-section of qualitative data, including various attitudes, perspectives, and subjective opinions. The nature of these data facilitated the use of textual narrative synthesis to process the key findings extracted in the review. To this end, the review synthesised three key subheadings. They include knowledge attitude and perception regarding menstruation among adolescent girls attending secondary schools in Nigeria.

#### 3.4.1. Knowledge of Menstruation and Menstrual Hygiene including Sources of Information Regarding Menstruation

A review of the studies included showed varying results about menstrual health. Knowledge about this biological process was limited to a few sources, and this was presented in the equally limited perspectives held by the participants [18]. For instance, most of the sampled participants (75%) in this study revealed that menarche (the first period) came with a heightened level of fear and embarrassment. It was revealed that only a minority of the girls sampled (47%) knew what menarche was and they experienced it with calmness [18]. In the same vein, only 22.6% of the participants in the study knew that menstrual blood came from the uterus, while 55.5% of the participants also failed to recognise the normal length of the menstrual cycle [37]. The majority of participants (96.42%) were aware of menarche before experiencing menstruation, with the primary source of this knowledge being their mothers (41.83%). The responses of their participants were presented, with one of them stating.

*“Upon my initial encounter with menstruation, I found myself perplexed and questioning its nature. I experienced apprehension in disclosing this information, uncertain of how others could respond, potentially including ridicule or similar reactions”.*

[28]

Contrasting results were presented with most of the participants (83.5%) revealing at least basic knowledge of menstrual hygiene [38]. A more in-depth study was performed, differentiating between participants in the rural setting against those in the urban setting. The authors demonstrated a notable disparity in knowledge of menstruation and menstrual hygiene between rural and urban areas, with participants from rural areas exhibiting much lower levels of understanding [19].

Based on the findings of this study, it was observed that mothers and other immediate family members played a vital role in providing information about menstruation hygiene for both rural (80.5%) and urban (72.5%) areas. One of the participants interviewed also provided similar findings.

*“Subsequently, I informed my mother regarding the matter, to which she responded by instructing me on the proper use of menstrual pads, demonstrating the appropriate technique”.*

[28]

Similarly, a majority of the studies observed that the primary sources of information about menstrual health were mothers and grandmothers [6,9,20]. Other authors also discovered the invaluable role played by school teachers to further educate girls about the basics of menstrual health [21]. It was revealed that 264 respondents were aware that genital tracts should be washed and had good knowledge of menstrual hygiene [23]. Some 59.7% of respondents did not know the causes of menses, channels through which menses flow, and intervals between menses. Only 39.7% of participants had basic knowledge about menses [32].

It was indicated also that respondents had good knowledge of the information of menstruation from their mothers (85.24%) and had low knowledge of the need to dispose of pads in bins (34.42%) [20]. It was demonstrated that students in high school had a lack of substantial understanding of menstruation cleanliness [21]. It was reported that knowledge regarding menstruation among rural-based adolescent female students (230, 56.7%) was significantly poor compared with the urban-based respondents (253, 42.2%) [19]. Data on menstruation were collected from the mothers of urban respondents (*n* = 435, 72.5%) and rural respondents (n = 327, 80.5%). A total of 407 adolescents (67.8%) attending urban schools and 318 adolescents (78.3%) enrolled in rural schools reported receiving information on menstrual hygiene from their mothers. Out of the total sample size of respondents, 68 individuals (constituting 16.74%) from rural schools exhibited a lack of knowledge regarding menstruation, whereas a smaller proportion of 20 respondents (equivalent to 4.0%) from urban secondary schools showed a similar lack of understanding [19].

#### 3.4.2. Attitude towards Menstruation and Menstrual Hygiene

The studies included showed varying perspectives concerning menstrual health. On one hand, it explored more positive attitudes, observing that a girl’s first menstrual period marked a period of celebration for the family and greater society [31]. It was also maintained that a girl’s menarche presented the opportunity for additional advice about womanhood [28]. These findings were the same, with one of the participants in that study explaining that:

*“No, I did not anticipate the occurrence and was there at my educational institution when the incident transpired. Upon observing the emergence of blood, I emitted vocal expressions of distress. According to my mother, I underwent a process of growth and development as a female child, resulting in my maturation”.*

[28]

Another participant in the study also showed that the onset of the first menstrual cycle also kick-started conversations about maturity, with girls who underwent the process being introduced to reproductive health education, while also being warned against being pregnant.

*“I experienced a significant level of fear and promptly informed my mother, who advised me to go with bathing and exercise caution in the event of physical contact, as it may potentially result in pregnancy”.*

[28]

In some of the studies, the onset of menarche was also seen to yield more negative sentiment from the male gender, with many girls expressing fear and restraining from revealing such biological developments to their fathers, brothers, and uncles. According to one participant, the male figures within the family setup also held some regressive beliefs, as follows.

*“No, I may choose not to disclose this information to guys. However, if they become aware, they express a preference for not being touched by a menstrual girl. Furthermore, they indicated that if I were to touch them, they would withhold payment. According to popular belief, women possess significant power and have the ability to deprive males of certain privileges or possessions, thereby prompting my decision to depart”.*

[28]

Furthermore, several respondents reported experiencing gestures of celebration from close family members following their marking of this phase and experiencing menarche. The authors confirmed that no specific ritual was enshrined in culture for girls experiencing menarche, but they still experienced kind, celebratory gestures mainly in the form of gifts [41]. More specifically, one participant explained that:

*“My mother exhibited a high level of enthusiasm, to the extent that she slaughtered a chicken and prepared jollof rice to cater to the entire household”.*

[31]

Regarding attitudes towards menstruation, it was reported that participants had negative attitudes towards menstrual hygiene [20]. Similarly, there was no significant positive attitude towards menstrual hygiene [21].

#### 3.4.3. Practice Regarding Menstruation and Menstrual Hygiene

Overall practice following menarche also saw girls transitioned into womanhood. In all the studies included, the time following the first menstrual period was denoted by girls receiving advice on what is expected of them following their maturity [6,20]. This period was also observed to generate a change in relationship dynamics with the opposite sex. It was explained that most girls received incomplete information about how they should relate with boys, which revolved around the importance of avoiding boys or being careful around them, and they practised poor menstrual hygiene [20,32]. In other instances, participants were found to have received exaggerated information to further drive the point home and drive them away from cutting relationship ties with the opposite sex. In one of the studies, a respondent shared:

*“Upon becoming aware of the situation, she assumed a seated position and proceeded to articulate statements designed to instil fear and dissuade me from engaging with individuals of the male gender. For instance, she posited that physical contact in the form of a hug with a male counterpart could result in an unintended pregnancy, among other related notions”.*

[31]

Most of the included studies also showed how most of the participants practised poor menstrual hygiene. It was reported that participants aged 10–14 years were more prone to maintaining poor practices related to menstruation compared to their counterparts in other age brackets [9]. Age was significantly associated with menstrual hygiene practices [34]. The study was to carry out a quasi-experimental design which saw the authors develop three experimental groups that were parent-led, peer-led, and a combination of the two, with the overall findings concluding that peer educators were highly effective in helping bring the greatest improvements toward menstrual health for girls after they experience menarche [31]. It was indicated that 55.8% of the population had good practices regarding menstrual hygiene. Some 33.9% of the respondents knew that the normal duration of a menstrual cycle is 27 days, while more than half (66.1%) indicated 28 days [38]. For menstrual hygiene practices, female adolescents indicated low practice of using sanitary pads during menstruation (68.8%) and changing at least three times a day (37.5%) [20]. There was also no significant practice of menstrual hygiene among respondents. The study concluded that respondents had a very low practice of menstrual hygiene [21]. For adolescents’ practice regarding menstrual hygiene, it was reported that practice of menstrual hygiene was reported by almost half (45.5%) of the respondents with half of secondary school girls of the ages of 10–14 years (46.8%), 15–19 years (45%), and 19 years and above (45%) indicating that they practise menstrual hygiene [41]. Poor menstrual hygiene practices were also reported among rural respondents compared with urban-based respondents (253, 42.2%) [19].

## 4. Discussion of Findings

Menstruation represents a significant milestone in pubertal development. However, a multitude of misconceptions surrounding this process often lead to inadequate knowledge and suboptimal hygienic practices among individuals. A comprehensive understanding of menstruation and its associated hygienic practices is crucial for the prevention of reproductive health issues. This systematic review focused on menstruation literacy and its practices among in-school adolescents in Nigeria. The stage of early adolescence marks a pivotal transitional phase during which gender norms can exert various influences on adolescents’ lives [45].

The results yielded in the previous chapter also presented an opportunity to broaden the discussion, and to achieve further gains in knowledge. Accordingly, the discussions chapter offered the opportunity to expand these discussions and compare them to the existing literature. To further guide this discussion, the study chose to structure the chapter according to the themes developed in Section 3.

Summary of major findings and Interpretation of the findings of included studies.

### 4.1. Knowledge of Menstruation and Menstrual Hygiene including Sources of Information Regarding Menstruation

Most of the studies included showed incomplete information, from a limited variety of sources about menstruation. In most cases, girls experienced menarche without being fully prepared, leaving them lost, surprised, and filled with fear [18,19,20,28,31,37]. This prevailing experience was also felt across other areas in the growing world. It was reported that developing nations persistently face a pervasive absence of availability when it comes to menstrual products and overall basic knowledge about this phase [46]. Moreover, many developing countries also experience insufficient access to educational materials and menstrual items such as feminine hygiene items, specifically sanitary pads or tampons. An occurrence of low knowledge was witnessed to still exist [47].

The review revealed a significant deficiency in menstrual knowledge among girls in rural regions before their first menstruation. Among the few who possessed any information, it typically came from their friends or mothers. This underscores the urgent need for providing health information, particularly concerning menstruation and hygiene, to girls from rural backgrounds.

It was observed that an overall lack of proper education about menstrual health within society also led to an increased prevalence of misconceptions, unhygienic practices, and taboos regarding menstruation, and most of the misconceptions mentioned were meant to recalibrate the relationships between the sexes, including the advancement of information that hugging could cause pregnancies. In the same breath, misconceptions were also noted that help advance avoidance intentions by boys to girls after they experience menarche, with many being convinced that contact with them could lead to bad luck [28,48].

The review also presented findings that showed progress in improving the knowledge base about menstruation and menstrual hygiene. It was evident that family ties played an important role in helping girls familiarise themselves with menstrual hygiene once they experienced their first period. It was reported that mothers and grandmothers were the main sources of information about these biological changes [49]. Family members of the opposite gender were seen to prefer to remain aloof to menstruation and reported some disparities did exist with access to such information [50].

Schools were also identified as an invaluable source of information about menstruation in most of the studies included in the review. This observation was consistent, and research that offered such educational institutions to standardise information burst many of the myths and misconceptions surrounding menstruation [37]. It reported similar results, explaining the effectiveness of public primary and secondary schools in providing a centralised point within which information about menstruation and menstrual hygiene could be shared, while also distributing sanitary towels [51].

The review also brought to light a high level of awareness among participants regarding menarche. Notably, the primary source of information was the respondents’ mothers. This finding aligns with a similar study conducted in Kaduna. Conversely, it contrasts with the results of a study conducted in South Africa which revealed mass media as the predominant source of information. The variance in these findings may be attributed to the educational background of the respondents’ mothers, as a significant proportion of them in this study were educated. Additionally, it has been noted that children are closer to their mothers, which can contribute to the prevalence of maternal influence as a source of information [52].

Most of the participants exhibited a decent degree of knowledge about menstruation and its corresponding hygiene practices. This conclusion aligns with previous research conducted in Abeokuta and the Netherlands, which also revealed a baseline level of adequate awareness regarding menstruation and menstrual hygiene at 70.2%. In contrast, the findings of a study conducted in Kebbi revealed that only 4% of participants had a satisfactory level of understanding of menstruation. This disparity may arise from differences in how knowledge was categorised in these studies, with some using broader categories like fair, good, and poor to assess knowledge levels [53].

### 4.2. Attitude towards Menstruation and Menstrual Hygiene

The findings of the review also showed contrasting results, with both positive and negative attitudes being observed among the respondents about menstruation. Overall, attitudes towards menstruation and menstrual hygiene in Nigeria and the rest of the continent were also found to vary significantly, according to different social, economic, geographic, and cultural contexts [54]. Accordingly, it was essential for policymakers and other stakeholders to identify such underlying contexts as the first step towards developing effective interventions [55]. More specifically, cultural beliefs were found to be a primary source for some of the main negative attitudes related to menstrual health. It was explained that some African cultures have traditional beliefs and taboos associated with menstruation [19]. Some communities view menstruation as being ritually impure, and this may lead to increasing restrictions about what girls can be allowed to take part in and what is prohibited [56]. It was reported that insufficient levels of education about menstrual health were also found to be prevalent in many developing countries, which led to negative attitudes towards menstruation. This knowledge gap was found to be a significant driver of many of the myths and misconceptions witnessed around the subject [20]. Addressing such knowledge gaps was also found to be necessary to be a key element to resolving most of the stigma and fear associated with menarche. It was explained that such stigma and negative attitudes are prevalent in some communities and this can lead to reluctance to discuss topics around menstruation and menstrual hygiene [57]. Evidence indicates that many organisations, both local and international, address attitudes regarding menstruation and menstrual hygiene challenges across the developed world [19,28]. Such institutions provide key benefits towards the empowerment of girls, through various actions such as provision of education, distribution of sanitary products, and the fighting of stigma. In the same way, such organisations also help with the advancement of policy, which guides governments to offer more support to ensure greater sensitisation about menstruation and menstrual hygiene. Findings from this review reveal that respondents exhibit negative attitudes. Most respondents experienced either fear or emotional distress during their first menstrual period. This pattern aligns with the findings of studies conducted in Bangladesh and Ethiopia [10]. These shared results across studies could be attributed to the inadequate provision of information and a lack of proper preparation for this natural process among the affected individuals.

### 4.3. Practices of Menstruation and Menstrual Hygiene

The onset of menstruation for girls in Nigeria and greater Sub-Saharan Africa was identified as an important milestone for those who experienced it directly, as well as the rest of the society. In most communities, this event was identified as a significant rite of passage for those involved, where older women who shared a close relationship with participants were expected to play a mentoring role [58]. In this capacity, older generations were seen to celebrate and practise such transitions amongst younger girls as a significant milestone. However, the incidence of incomplete information was also found to be detrimental to the overall society, mainly with regards to helping advance misconceptions and myths within their immediate society. Mainly, such myths were propagated to ensure that young girls were discouraged from maintaining friendly relationships with members of the opposite sex.

The review findings reported that most participants showed poor menstrual hygiene practice, which was prevalent in many states across the developed world. Some of the included studies showed that younger girls were more likely to practise poorer menstrual hygiene following the onset of menarche and such occurrences were mostly associated with them being recipients of insufficient information to prepare them for this new phase. Overall, poor information sharing and sensitisation also saw many young girls facing challenges with the disposal of used sanitary towels and poor washing and cleaning of genitals. It was revealed that many societies did not advise girls that used sanitary pads should not be seen by anyone and should instead be put in a garbage bin or wrapped in black polythene before being disposed of [46,59]. It was reported that, on the contrary, few members of the community were interested in showing adolescents how to dispose of sanitary pads, which was considered an acceptable method of disposal following their use [60].

Adolescents must prioritise adherence to appropriate menstrual hygiene practices to effectively mitigate the risk of contracting illnesses. The utilisation of recyclable items for menstrual hygiene poses a heightened risk of getting infected if not properly cleaned and dried. The results of this review revealed that a significant proportion of participants utilise cloth/rag and toilet paper as menstrual hygiene products. This phenomenon is consistent with the findings of research conducted in Owerri, Nigeria, which also uncovered the utilisation of unhygienic absorbent materials during the menstrual period. The great frequency of this practice can be related to the significant levels of poverty, as a considerable proportion of the respondents’ parents and guardians are not engaged in gainful employment. It is imperative to acknowledge that effective menstrual hygiene practices entail the safe and hygienic disposal of absorbent materials utilised during the menstrual cycle [21].

In this review, it was shown that a significant number of participants discarded these sanitary items without proper packing and proceeded to flush them down the toilet. This practice was found to be inconsistent with the results of earlier research wherein it was claimed that a significant proportion of the participants either incinerated or encased the absorbent materials before disposal. The act of disposing of these items by refraining from wrapping and then flushing them down the toilet is not only aesthetically displeasing but also has the potential to foster the proliferation of insects and vermin, a phenomenon that should be actively prevented [18].

Menstrual hygiene practices observed in this review were very poor, with only 15% of respondents adhering to good hygiene practices. This figure is lower than the rates reported in studies conducted in Ghana and Northwestern Nigeria, where the percentages were 40.9% and 58.7%, respectively. This suggests that having good knowledge about menstruation and menstrual hygiene does not necessarily translate into proper practice [20].

### 4.4. Strengths and Limitations of the Review

This systematic review offered a comprehensive overview and in-depth assessment of existing research on menstruation and menstrual hygiene among in-school adolescents in Nigeria. With extensive research carried out, this is the first systematic review to be conducted on this topic in Nigeria. This review enhances clarity due to the extensive body of published material identified and improves the overall applicability and uniformity of findings.

Moreover, this analysis provides significant insights into the awareness, attitudes, and practices about menstruation and its hygiene among teenagers attending schools in Nigeria. The review had an in-depth inquiry into the topic.

The review had some limitations, which included language bias where only articles published in English language were included. Current publications were omitted due to a lack of full-text article exclusion, and there was no double-checking of extraction of data carried out by the first author only. Another limitation regarding this review was the exclusion of out-of-school female adolescents, which has led to limited information regarding girls that might be absent from school.

## 5. Conclusions

The systematic review conducted indicated various gaps in knowledge about menstruation and menstrual hygiene among the different geographical zones in Nigeria. The results of the review indicated that a significant proportion of the participants in Nigeria were not adequately prepared for the onset of their first menstrual period. Teenagers exhibited a limited understanding of menstruation. Attitudes towards menstruation and menstrual hygiene also varied: most attitudes were low. In terms of attitudes, the female students generally exhibited negative attitudes across various items related to menstrual hygiene. Notably, the only exception was their positive attitude towards using water and soap to wash their hands during menstruation.

The review shows a significant gap between adolescents’ understanding of menstruation and menstrual hygiene and actual hygienic practices during menstruation. To address this, concerted efforts are required to have educational programmes to educate adolescents comprehensively on the significance of menstruation and the value of upholding safe sanitation practices, in both educational settings and domestic surroundings. Furthermore, the study underscores the crucial role of parental support during the menstrual period. The results suggest a significant correlation between cohabitating with family and improved menstrual hygiene practices. Therefore, parents need to offer the requisite assistance and direction to youngsters at this pivotal period.

Being a novel work, the contribution of this article included identifying knowledge gaps and summarising the current state of knowledge, attitudes, and practices regarding menstruation among in-school adolescents in Nigeria. It also identified some challenges faced by in-school adolescents relating to menstruation which included poor menstrual hygiene practices and low access to menstrual products. This article further provided information on the prevalence and patterns of menstruation among female in-school adolescents. It also provided information on recent effects of interventions aimed at improving menstrual health among adolescent girls, which included education initiatives and access to menstrual hygiene products. Our findings are expected to serve the need for educational intervention for other regions in Africa and for incorporating comprehensive menstrual health education programmes in secondary schools.

The relevance of the publication includes the public health impact and significance of understanding menstrual health among in-school adolescents. It also provides information assisting other researchers to develop new ideas which would help to shape the foundation of writing more articles regarding menstruation.

### Recommendations for Future Research/Practice/Policy

The suggestions proposed in this systematic review are as follows, by the findings.

1. Standardisation of sensitisation practices across public and private primary and junior secondary schools for teenage girls about menstruation and menstrual hygiene. Such actions promise improvement of interventions against many of the myths advanced about menarche and the onset of menstruation for girls.
2. Additional efforts should be placed towards enlightening girls about proper methods of hygiene and menstrual health once they reach this phase. Such actions would help better secure the overall reproductive health of young girls, while also helping them address many related concerns, such as how to properly dispose of used pads and tampons.
3. Governments and other non-governmental institutions within the country can also partner more closely to ensure enhanced availability of menstruation supplies, including pads and tampons, amongst more underprivileged sections of the population. By working together more closely, such institutions can improve their reach effectively.
4. The prevalence of negative attitudes suggests a lack of comprehensive understanding among the respondents in the study area. Future research endeavours should prioritise the teaching and enlightenment of teenage girls and women about the significance of menstrual hygiene.
5. A notably deficient level of knowledge and awareness regarding the disposal of menstrual waste, particularly the notion that a dustbin is not required, was indicated. Schools must implement educational programmes aimed at sensitising and empowering students, both girls and boys, about menstruation. These programmes should address the challenges related to menstrual hygiene, including waste collection, segregation, storage, and disposal, as well as addressing taboos associated with menstruation and the potential blockages of toilets due to the disposal of used napkins.

## Figures and Tables

**Table 1:** Search strategy tool (SPIDER).

**S** stands for Sample	Adolescents going to schools in Nigeria
**PI** stands for Phenomenon of Interest	Menstruation literacy, menstruation hygiene
**D** stands for Design	Questionnaire, intervention, interview, focus group discussions (FGDs), survey
**E** stands for Evaluation or outcome	Knowledge of menstruation and menstrual hygiene, sources of information regarding menstruation, attitude towards menstruation, perception of menstruation, practices, and beliefs of menstruation hygiene
**R** stands for Research type	Quantitative, cross-sectional, quasi-experimental, qualitative

**Table 2:** Search record/log.

Date	Database	Keywords	Strategy	Results (Hits)	Refine	Notes
29 May 2023	PubMed	Adolescent OR girl	Use of Boolean OR	200	Last 5 years and peer-reviewed journals = 50	7 articles were useful. 3 reviews of literature. 3 systematic reviews. Key references added.
6 June 2023	EMBASE	Youth OR young	Use of Boolean OR	150	Last 5 years and peer-reviewed journals = 40	5 articles were useful. 2 reviews of literature. 2 systematic reviews. Key references added.
9 June 2023	EBSCO	Menstrual health literacy OR Information	Use of Boolean OR	135	Last 5 years and peer-reviewed journals = 30	7 studies looked useful. 3 reviews of literature. 3 systematic reviews. Key references added.
15 June 2023	CINAHL Plus	Attitude AND Practice	Use of Boolean AND	140	Last 5 years and peer-reviewed journals = 25	5 studies looked useful. 2 reviews of literature. 1 systematic review. Key references added.
10 July 2023	BioMed Central	Schoolgirls AND in-school adolescents	Use of Boolean AND	300	Last 5 years and peer-reviewed journals = 15	6 articles were okay. 3 reviews of literature. 1 systematic review. References added.

**Table 3:** Inclusion and exclusion criteria.

	Inclusion	Exclusion
Sample (S)	Adolescents (age 10–19) attending secondary schools in Nigeria	Adolescents (age 10–19) not attending secondary schools in Nigeria
Phenomenon of Interest (PI)	Menstruation, menstrual literacy, menstrual hygiene	Articles that did not research menstruation, menstrual literacy, menstrual hygiene
Design (D)	Questionnaire, intervention research, interviews, focus-group discussions, survey	Case study, cohort study, randomised control trials (RCTs)
Evaluation (E) outcome	Knowledge of menstruation and menstrual hygiene, sources of information regarding menstruation, attitude towards menstruation, practices, and beliefs of menstruation hygiene	Articles that did not measure outcomes regarding knowledge of menstruation and menstrual hygiene, sources of information regarding menstruation, attitude towards menstruation, practices, and beliefs of menstruation hygiene
Research type (R)	Primary research, peer-reviewed, quantitative studies, qualitative studies, cross-sectional studies, quasi-experimental studies that had a component of menstrual information, studies that included adolescents up to the age of 19, no timeframe, published in English language	Not peer-reviewed, articles that are not primary research, systematic reviews, commentaries, letters to editors, short communications, studies aimed at adult women

**Table 7:** Data extraction table (the characteristics of the included 15 articles and summary of findings).

Reference	Study Setting	Study Design, Sample Size	Aim	Study Findings	Limitations
Tomlinson 2022 [28]	Suburb settlements located in Benin City in Edo State, Nigeria	Mixed methods (qualitative and quantitative, such as in-depth interviews and questionnaires). 60 girls for the interview and 600 girls for the questionnaire (between 11 and 19 years).	To elucidate the knowledge, attitudes, and practices about menstruation among young female students.	The mean age of onset of menarche was found to be 13.1 years, with the prevailing symptoms reported as abdominal discomfort in 73.7% of cases and mood irritation in 40.7% of cases. Knowledge about menstruation was obtained from their mothers. 76.9% of participants answered correctly when basic questions were asked. 42.6% of the girls had anxiety for their next period. 4.7% of respondents had adequate menstrual practice at school which included the use of sanitary pads and sanitation facilities.	No longitudinal data for causal inference. Selection bias when selecting only in-school adolescents as outcomes may not reflect the broader population. Information bias as respondents were too scared to ask for clarification of questions. Exposure or outcome misclassification. Social desirability bias by girls exaggerating negative experiences. Only adolescent girls were used and the influence of other counterparts such as males and teachers was not considered.
Adegbayi 2017 [31]	Redeemer’s University, Ede, Osun State, Nigeria	Quantitative method using questionnaires and qualitative method using in-depth interviews. 136 undergraduate female students aged between 16 and 25 years.	To investigate the many sources of menstrual knowledge and the practices used throughout the menstrual cycle.	Most respondents (95%) reported that they obtained knowledge about menstruation from their mothers, female relatives, and school classes before experiencing their first menstrual period.	The study’s representativeness of the entire population may be limited due to its exclusive selection from a private university, where most of the female participants are from the upper middle class and possess a higher socio-economic position.
Agbede and Ekeanyanwu 2021 [33]	Four secondary schools in Ogun State, Nigeria	Quasi-experimental using an educational intervention where data were collected using a researcher-structured questionnaire. 120 in-school adolescent girls.	To ascertain the impact of a training programme on menstrual hygiene practices.	At the end of the intervention, menstrual hygiene practice levels increased.	The study’s conclusions were derived from an examination of traditional and cultural variables that could potentially contribute to increased representation of the Nigerian populace.
Bolanle, Ayoade and Sola 2021 [37]	Lagelu Local Government Area in Oyo State, Nigeria	Descriptive cross-sectional study using a semi-structured questionnaire. A total of 421 female adolescents between the ages of 10 and 19 years old were selected.	To examine the extent of awareness and adherence to sanitary menstrual practices among adolescents.	Approximately 50.8% of individuals exhibited a satisfactory level of understanding regarding the topic of menstruation. The study revealed a significant lack of hygiene knowledge, with just 22.6% of participants demonstrating an accurate understanding that monthly blood originates from the uterus. Additionally, most participants (55.5%) were unaware of the typical duration of the menstrual cycle. A total of 42.2% of the participants indicated their preference for utilising washable and reusable materials.	Most community settings do not have infrastructures for safe and private menstrual hygiene and the ones that are present are underequipped or mismanaged.
Ekoko and Ikolo 2021 [9]	Rural community secondary schools in Delta State, Nigeria	Cross-sectional descriptive study. 471 female students between the ages of 10 and 19.	To enhance the level of menstrual hygiene awareness and foster positive attitudes regarding menstruation hygiene and periods among secondary school girls residing in rural parts of Delta State, Nigeria.	A significant proportion of teenagers, specifically 290 individuals (61.5%) and 369 individuals (78.3%), respectively, demonstrated a lack of knowledge of the concept of menstruation and its underlying causes. Out of the whole sample population, it was observed that 213 individuals, accounting for 45% of the female participants, initially utilised a washable cloth as a means of managing menstruation.	Adolescents were not ready for the menstruation experience which resulted in limited knowledge of how to use and dispose of absorbent materials.
Fehintola et al., 2017 [18]	Public secondary schools in Ogbomoso North Local Government Area (LGA) of Oyo State, Southwest Nigeria	Cross-sectional study. 447 respondents between the ages of 10 and 19 were selected.	To evaluate the level of understanding, attitudes, and behaviours about menstruation and menstrual hygiene.	Most participants (96.42%) were aware of menarche before experiencing menstruation, with the primary source of this knowledge being their mothers (41.83%). Most of the participants (55.92%) had a satisfactory level of understanding regarding menstruation and menstrual hygiene.	The causal conclusion is limited due to the study being cross-sectional. Information bias as the study was based on self-reported information on menstruation.
Obande-Ogbuinya et al., 2022 [23]	Secondary school girls in Ikwo local government area, Ebonyi State, Nigeria	A cross-sectional descriptive study using an administered questionnaire. 315 participants were recruited for the research.	To determine the knowledge and attitude toward menstrual hygiene practices among adolescents.	Of most teenage girls, 266 (84.4%) indicated that they changed sanitary pads after 6 h and 269 (85.4%) were positive that the genital tract should be washed with water during menstruation. 264 Respondents (83.8%) were aware that genital tracts should be washed from front to back during menstruation, and 259 (83.2%) of the adolescent girls indicated that sanitary pads should be disposed of immediately after use.	Cause-and-effect relationships might not be shown between study variables due to the nature of the study, which is cross-sectional.
Okafor-Terver & Chuemchit, 2017 [32]	Secondary schools in Katsina State, Nigeria	Cross-sectional survey. 395 female adolescents between the ages of 10 and 19.	To assess the knowledge, beliefs, and practice of menstruation among in-school adolescents in Katsina State, Nigeria	59.7% of respondents did not know what causes menses, the channels through which menses flow, and the duration of time between menstrual cycles. Only 39.7% of participants had basic knowledge about menses. Also, 62.3% use commercial pads; 10.8% and 26.9% use tissues and cloths. 86% wash their rags with water and soap and 35% dry them under the sun while others dry them under the bed or in their room.	The use of Hausa, which is the local language, to design the questionnaire and the enthusiasm of the Muslim conservative girls to take part in the research, who are conservative in nature. The study cannot be generalised in Nigeria and other countries.
Buradum, Etor and Edison 2020 [38]	Secondary schools in Khana, Rivers State, Nigeria	Descriptive cross-sectional survey design using a questionnaire. 250 female students were recruited for the study.	To examine knowledge of menstrual hygiene practices among female secondary school students.	Findings indicated that 55.8% of the population had adequate knowledge of menstrual hygiene. Approximately 33.9% of the participants possessed knowledge regarding the typical length of a menstrual cycle, which is 27 days, while more than half (66.1%) indicated 28 days.	There was no proper explanation on how to calculate the menstrual cycle, which could be of help to the respondents.
Gorah, Haruna and Ufwil 2020 [20]	Secondary schools in Bokkos Local Government Area, Plateau State	Survey research design. 325 female adolescents	To examine the knowledge, attitude, and practices of female students regarding menstrual hygiene.	Respondents had good knowledge of the information about menstruation from their mothers (85.24%) and had low knowledge of the need to dispose of pads in bins (34.42%). Participants had negative attitudes towards menstrual hygiene.	Absenteeism of girls from school. No generalisability of results.
Ibeagha 2022 [21]	Public secondary schools in Akinyele Local Government Area of Oyo State, Nigeria	Descriptive research design. 1200 female junior and senior secondary school adolescents.	To investigate female adolescents’ knowledge, attitudes, and practices regarding proper menstrual hygiene and menstruation.	The findings indicate a lack of substantial understanding regarding menstrual hygiene among secondary school students (*p* > 0.05). Furthermore, there is a notable absence of positive attitudes towards menstrual hygiene and a lack of adherence to menstrual hygiene practices.	The study was conducted within a single state, hence constraining the generalisability of its findings. Relevant conclusions cannot be established as well.
Okeke et al., 2021 [41]	Secondary schools in Owerri Municipal Council of Imo State, Nigeria	Descriptive survey research design using a structured validated questionnaire. 420 female adolescent girls.	Menstrual hygiene practices were examined among in-school adolescents in Owerri, Imo State, Nigeria.	Menstrual hygiene practice was reported by almost half (45.5%) of the respondents. Additionally, it was found that a significant proportion of female students in secondary school engage in menstrual hygiene practices. Specifically, 46.8% of girls aged 10–14, 45% of girls aged 15–19, and 45% of girls aged 19 and above reported practising menstrual hygiene.	The population was from the urban area of the state, which is not representative of the rural population.
Edet et al., 2020 [19]	Eight secondary schools were selected from two Local Government Areas (urban and rural) in Cross-River State, Nigeria	Exploratory descriptive cross-sectional design. 1006 female students aged between 10 and 18 years.	To determine menstruation and menstrual hygiene knowledge among secondary school adolescents as a step to plan an appropriate health promotion intervention.	Among the participants, it was observed that a majority of rural-based teenage female students (56.7%) exhibited a notable deficiency in their understanding of menstruation and menstrual hygiene practices, in contrast to the urban-based respondents (42.2%). Data on menstruation were collected from the mothers of urban respondents, with a total of 435 individuals representing 72.5% of the sample. Similarly, for rural participants, data were received from the mothers of 327 individuals, accounting for 80.5% of the sample. Furthermore, it was shown that most teenagers were in both urban schools (67.8%).	The results obtained from this research are limited in their applicability to Cross-River State and cannot be extrapolated to other states within Nigeria. The subject matter of bias in response pertains to a delicate societal matter within the field of research.

## Data Availability

Not applicable.
